# Needle-knife papillotomy for removal of impacted fully covered self-expandable metal stent

**DOI:** 10.1016/j.igie.2025.03.002

**Published:** 2025-03-26

**Authors:** Pang Yong, Wang Tao, Zhang Hui, Luo Zhu-lin, Xie Chuan, Tian Fu-zhou

**Affiliations:** Department of General Surgery, The General Hospital of Western Theater Command, Chengdu, Sichuan, China

Endoscopic retrieval of a proximally migrated biliary metal stent is technically challenging and occasionally ends in failure.^1^ Whereas several retrieval techniques have been published,^2^^,^^3^ we report an innovative approach with the use of a needle-knife papillotomy for successful removal of an impacted fully covered self-expandable metal stent (FCSEMS).

A 61-year-old woman was admitted to our hospital for scheduled removal of a previously placed biliary stent. The patient’s medical history included right hemihepatectomy for intrahepatic bile duct stones 1 year earlier, followed by the development of obstructive jaundice due to benign stenosis of the hilar bile duct 6 months postoperatively. Four months earlier she underwent endoscopic retrograde cholangiopancreaticography (ERCP) with placement of an FCSEMS, and effective biliary decompression was achieved .

During repeated ERCP 4 months after stent placement, the distal end of the FCSEMS was found to have migrated internally into the papilla (Fig. 1). Despite the use of multiple devices, including balloons, oval snares, rat-tooth forceps, and changes in duodenoscope position and torquing maneuvers, it was impossible to remove the FCSEMS. Finally, a plastic stent was placed inside the FCSEMS.

**Figure 1:**
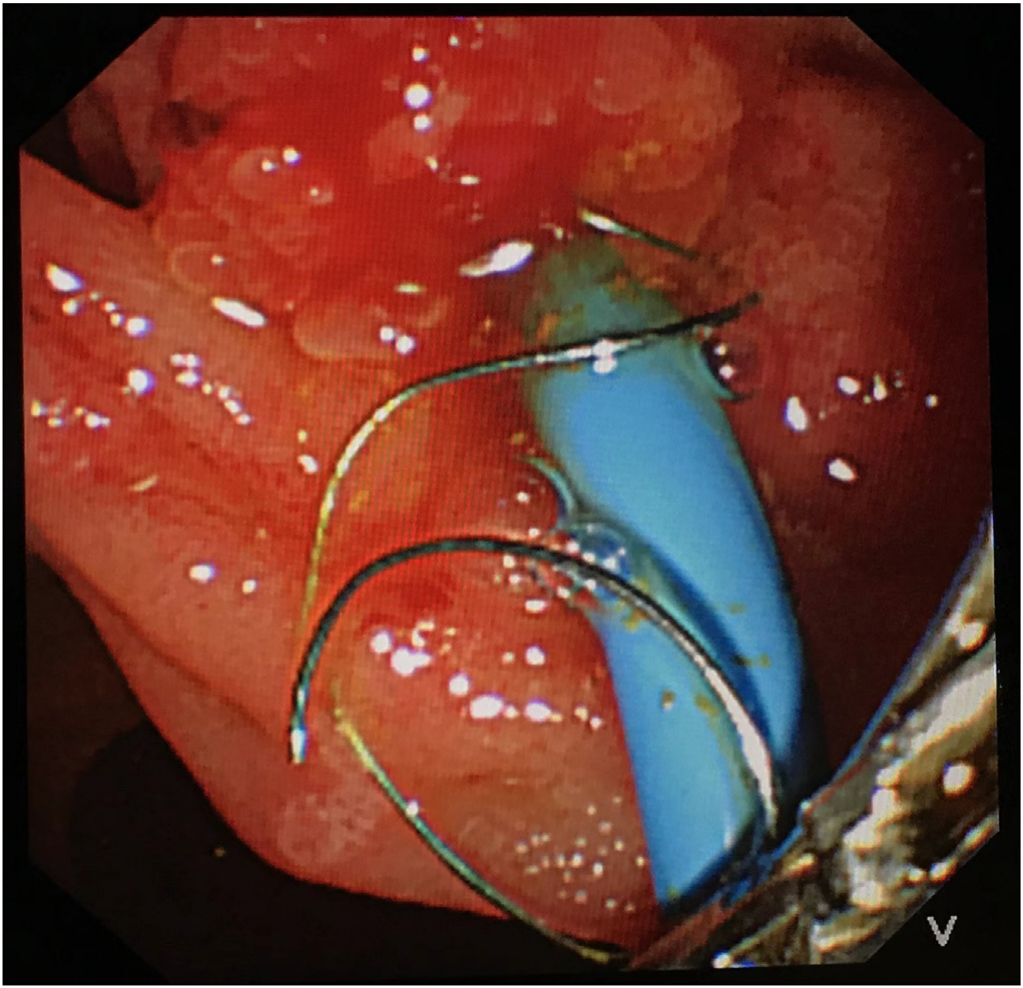
Endoscopic image showing biliary plastic stent inside a fully covered self-expandable metal stent that migrated proximally into the bile duct; forceps grasp the visible end of the stent.

To avoid open surgery as much as possible, we carefully designed a plan for the next endoscopic intervention. We first confirmed on an external model of pig liver that the needle-knife would not generate excessive heat when touching the FCSEMS. Then a decision was made to perform needle-knife papillotomy to extract the impacted FCSEMS 7 days later. The electrosurgical current setting was the same as that used in routine papillotomy. The wire was then extended 5 mm from the tip of the catheter and inserted into the papillary orifice (Fig. 2). A 7- to 8-mm incision was made at the 12-o'clock orientation. The proper orientation for the cut was achieved by creating pressure with the cutting wire on the roof of the papilla, either the elevator or the control knobs being used to deflect the wire. After the mucosal and submucosal layer were precut, the metal mesh of the FCSEMS could be seen. With gradual exposure, the distal end of the FCSEMS was completely excavated along its edge by the needle-knife (Fig. 3). Then the inner plastic stent was removed, and a snare was used to grab the distal end of the FCSEMS (Fig. 4). Constant traction was applied by pulling and torquing the duodenoscope for about 1 to 2 minutes (Fig. 5). The FCSEMS was retrieved completely (Figs. 6 and 7). There were no postprocedural adverse events. The patient was doing well 1 month later.

**Figure 2:**
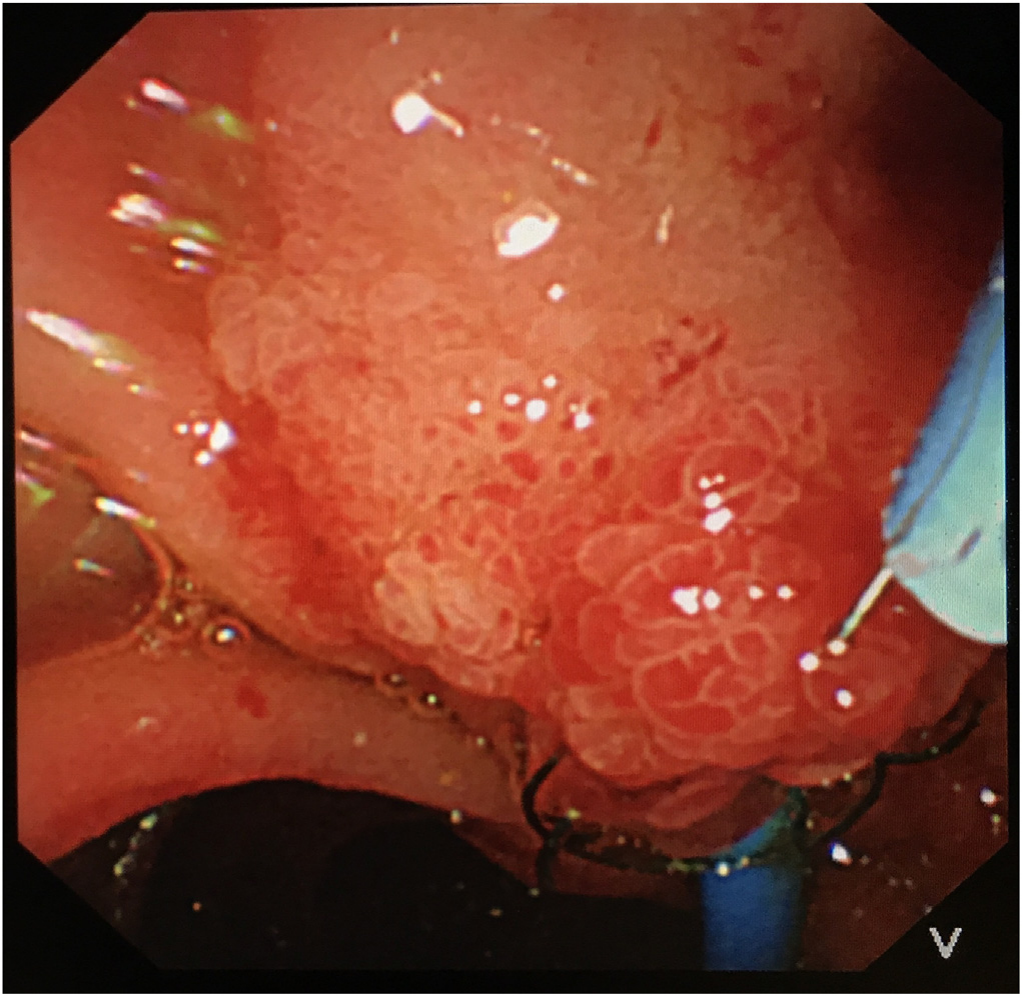
Needle-knife extended from the catheter and ready to perform needle-knife papillotomy to remove impacted metal stent.

**Figure 3:**
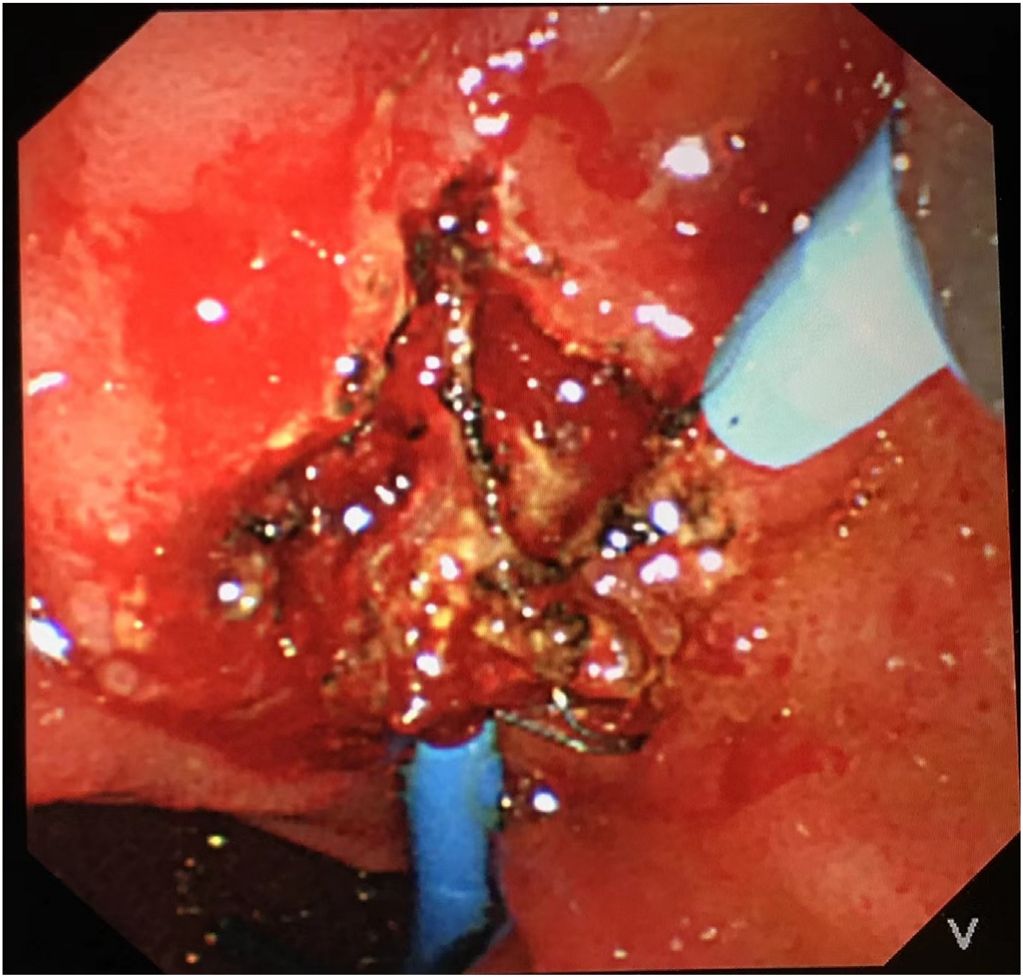
Needle-knife papillotomy to expose distal end of metal stent.

**Figure 4:**
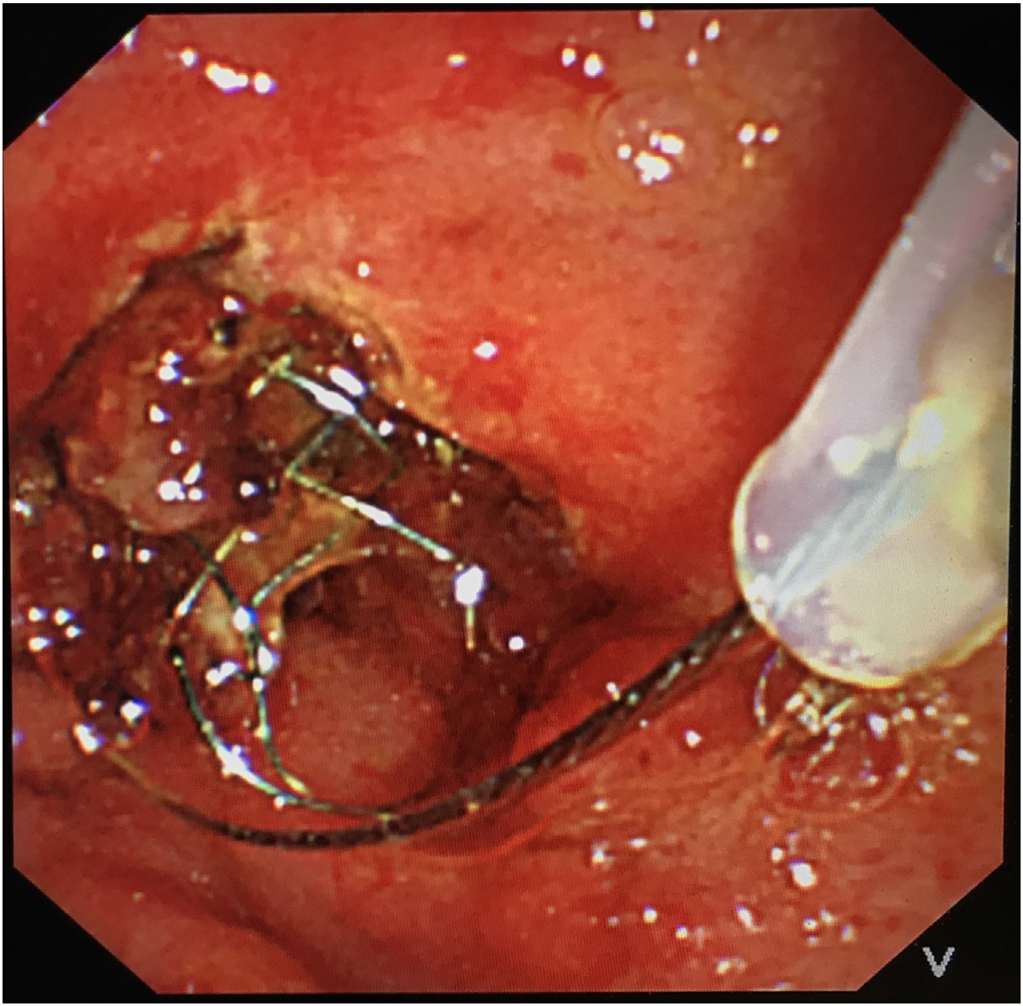
Use of a snare to grab the exposed distal end of the metal stent.

**Figure 5:**
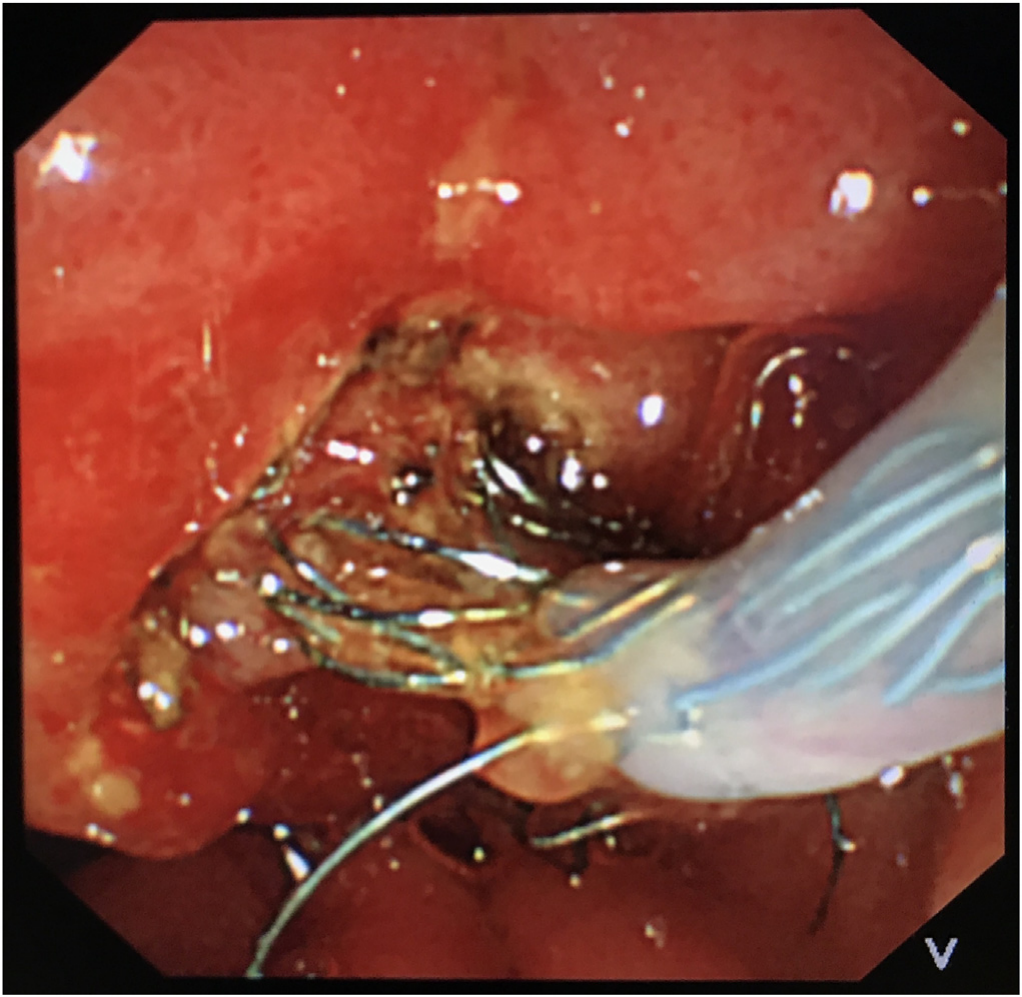
Distal end of metal stent grabbed and pulled out by use of snare.

**Figure 6:**
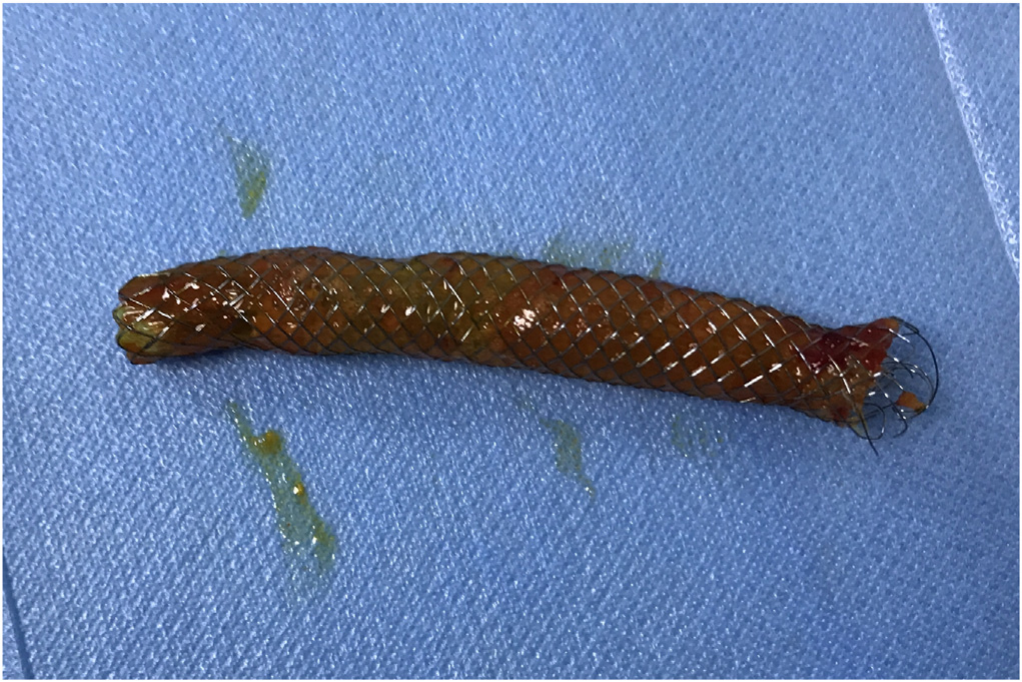
Close-up of completely retrieved fully covered self-expandable metal stent.

**Figure 7:**
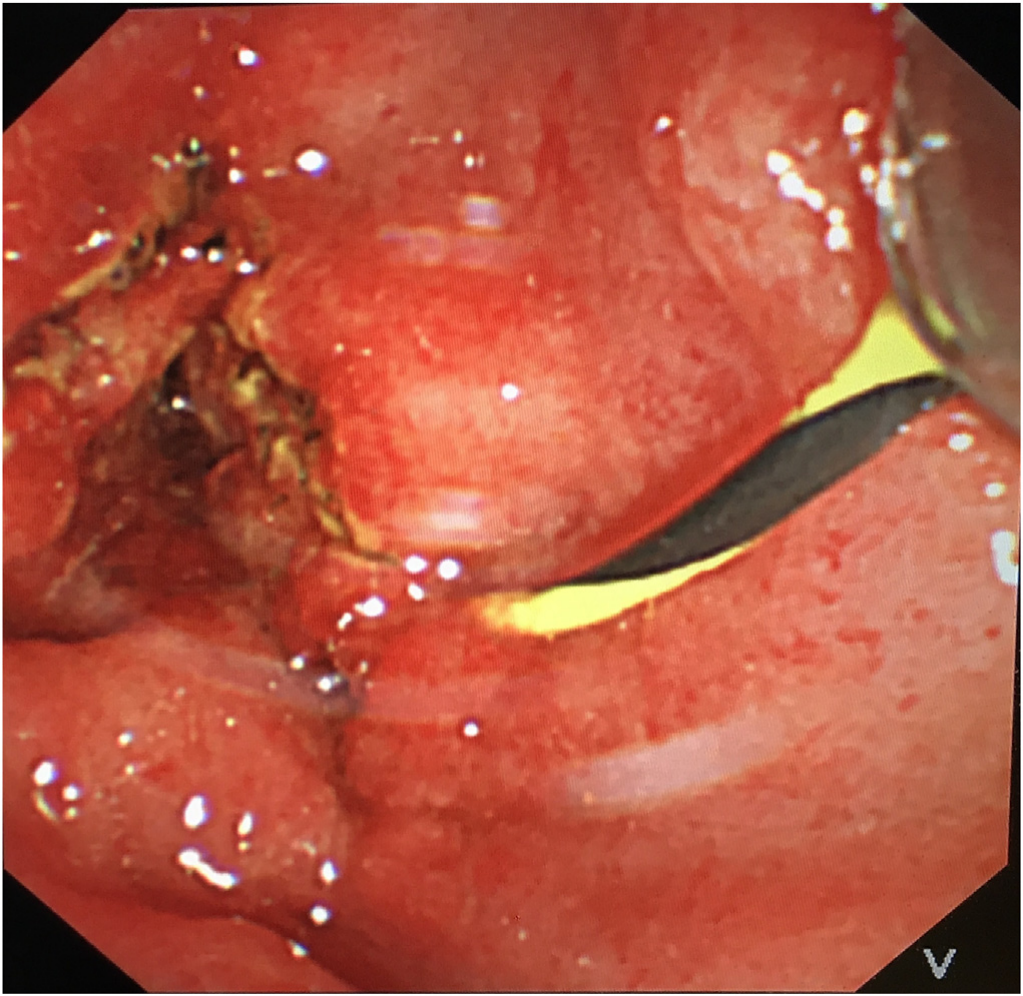
Appearance of papilla after removal of fully covered self-expandable metal stent.

In this case, we describe the use of needle-knife papillotomy to enhance exposure for the retrieval of an internally migrated covered biliary metal stent. Whereas needle-knife papillotomy is commonly used to access the common bile duct during challenging ERCP procedures or for the removal of impacted stones at the papilla, its application to facilitate the removal of a covered metal stent may represent a novel approach.

## Disclosure

All authors disclosed no financial relationships.
